# Supplementary material for: Improved Conductivity in Gellan Gum and Montmorillonite Nanocomposites Electrolytes
Source: Molecules. 2022 Dec 9;27(24):8721. doi: 10.3390/molecules27248721 (PMC9785073; doi:10.3390/molecules27248721)
Supplement: Supplementary file 1 [file molecules-27-08721-s001.zip › molecules-2022632-supplementary.pdf]

Supplementary Material

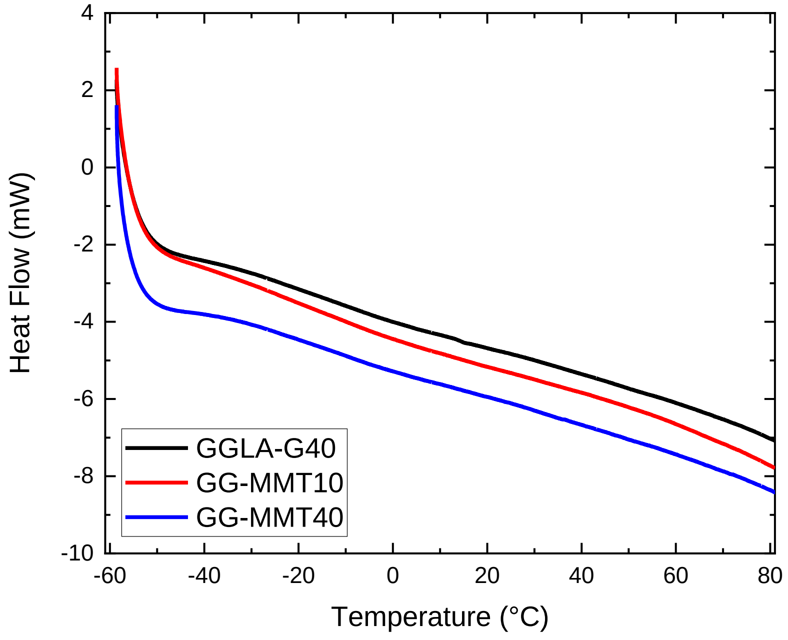

Figure S1. DSC plots of GGLA-G40, GG-MMT10, and GG-MMT40.

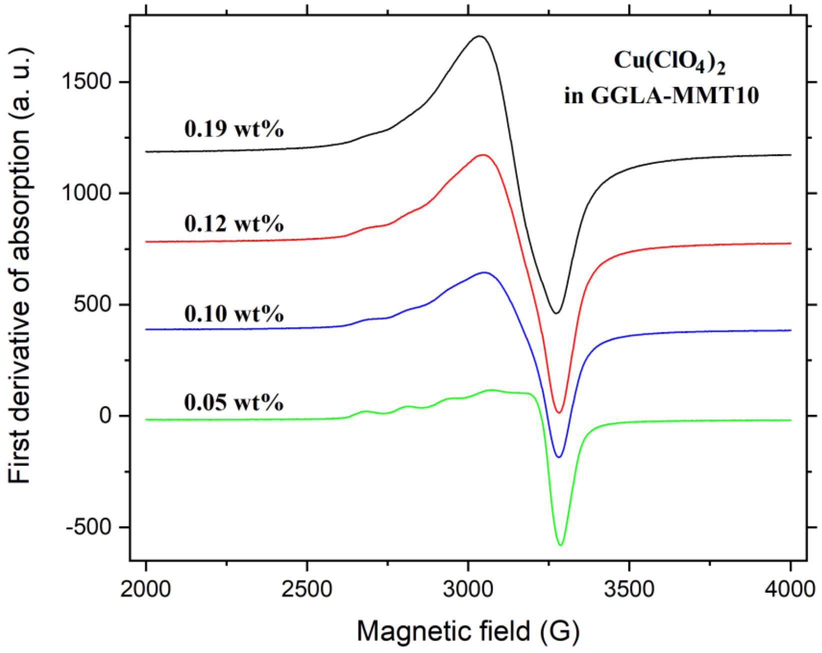

Figure S2. X-band CW-EPR spectra measured at ≈300K of GG-MMT10 samples at different $Cu(ClO_4)_2$ concentrations. All spectra were measured at the same experimental conditions and normalized to the sample mass. The microwave frequency: ≈ 9.43 GHz.

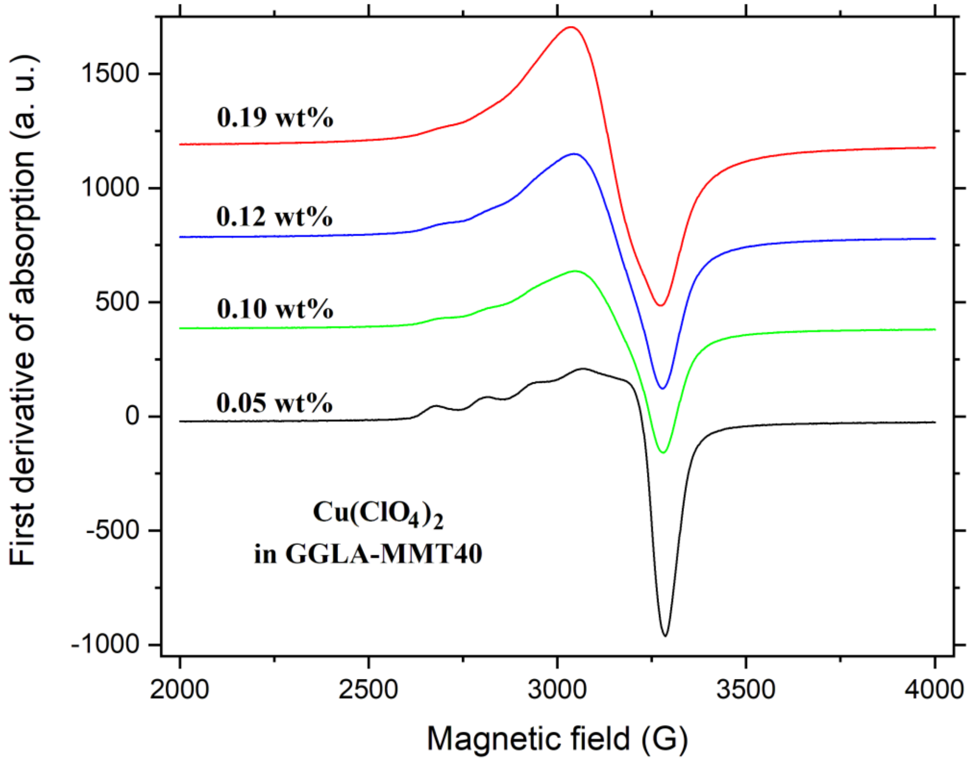

Figure S3. X-band CW-EPR spectra measured at ≈ 300K of GG-MMT40 samples at different $Cu(ClO_4)_2$ concentrations. All spectra were measured at the same experimental conditions and normalized to the sample mass. The microwave frequency: ≈ 9.43 GHz.
